# When, where and how does microbial community composition matter?

**DOI:** 10.3389/fmicb.2014.00497

**Published:** 2014-09-26

**Authors:** Diana R. Nemergut, Ashley Shade, Cyrille Violle

**Affiliations:** ^1^Institute of Arctic and Alpine Research, University of ColoradoBoulder, CO, USA; ^2^Environmental Studies Program, University of ColoradoBoulder, CO, USA; ^3^Department of Biology, Duke UniversityDurham, NC, USA; ^4^Department of Microbiology and Molecular Genetics, Michigan State UniversityEast Lansing, MI, USA; ^5^CEFE UMR 5175, CNRS - Université de Montpellier - Université Paul-Valéry Montpellier – EPHEMontpellier, France

**Keywords:** structure-function, biodiversity-ecosystem function, trait-based approaches, species-species interactions, ecological trade-offs, microbial community assembly, trait distributions

Our planet is experiencing rates of environmental change unprecedented in modern times, and an understanding of how microbes both mediate and respond to these shifts is an important research challenge (De Vries and Shade, 2013). Because of the temporal and spatial scales over which microbes function as well as their extreme diversity, dynamics in microbial structure and processes are typically examined at the community level. However, the factors that drive patterns in microbial structure and function, and the links between them, remain widely debated (Prosser et al., 2007). In this issue, such patterns in microbial communities are further documented for soils, lakes, streams and ocean provinces (Arnosti et al., 2012; Jones et al., 2012; King et al., 2012; Larouche et al., 2012). Additionally, the importance of spatial and temporal dynamics (Armitage et al., 2012; Arnosti et al., 2012; Jones et al., 2012; Larouche et al., 2012) and interactions with macrobiota (King et al., 2012) in driving these patterns is demonstrated. Yet, a central but unanswered question is: “does knowing who is there help us to better understand what they are doing?” Indeed, as shown here by Salles et al. (2012), links between structure and function can often be weak, both at the level of the individual and at the level of the community. Several papers in this special issue, “The Causes and Consequences of Microbial Community Structure,” use empirical or modeling approaches as well as literature reviews to enrich our mechanistic understanding of the controls over the relationship between community structure and ecosystem processes. Specifically, authors address the role of trait distributions and trade-offs, species-species interactions, evolutionary dynamics, community assembly processes and physical controls in affecting “who's there” and “what they are doing.”

Trait-based approaches can provide mechanistic links between community structure and function, and are gaining popularity in microbial ecology (Krause et al., 2014). Importantly, the distribution of traits within a community may affect the relationship between structure and function (Webb et al., 2010). Thus, as highlighted in this issue by Comte et al. (2013), traits can be considered at both the individual and the community level, where trait distributions may have important implications for emergent properties (e.g., redundancy). Indeed, Shade et al. (2012) highlight a variety of traits that may govern the stability of individual organisms, populations and communities including plasticity, tolerance and dormancy. Folse and Allison (2012) used a multi-nutrient, multi-genotype model of enzyme activity, and showed that trait distributions could yield insight into the relationships between biodiversity and ecosystem function. They found that generalists dominated at low levels of community diversity when rates of enzyme production and enzyme diffusion were lowest. Matias et al. (2013) used a simple microcosm experiment and examined the response of assembled communities to fluctuations in salinity. Their results were somewhat different from Folse and Allison (2012), as they found that community diversity was positively related to productivity and that generalists were more productive and less variable over time. Their work also showed that there did not appear to be a fitness trade-off associated with generalization. Comte et al. (2013) took a novel approach to examine plasticity and redundancy in freshwater bacterioplankton communities, and described explicit metrics to track these traits within community transplant experiments. They showed that plasticity appeared to be an intrinsic community property while redundancy was affected by external environmental factors. Their work also revealed strong relationships between community plasticity and redundancy, with no evidence for trade-offs and a possible co-selection of these attributes.

As well, species-species interactions can affect the relationship between communities and processes. In the model presented by Folse and Allison (2012), the importance of both “coalitions” of complementary organisms and the abundance of “cheaters,” or organisms that use a public good without contributing to its production, increased under high levels of enzyme production. They also found that the presence of cheaters could affect the relationship between biodiversity and function. Fox (2012) offered a cautionary tale in terms of our ability to interpret relationships between abundance and “adaptedness” because of organismal interactions. He used a consumer-resource model to demonstrate that, at medium levels of niche overlap, outcomes of competition can be unpredictable, decoupling relationships between abundance and adaptation.

Evolutionary dynamics can also alter relationships between structure and function. In a Perspectives Article, Choudoir et al. (2012) advocate for population-level approaches to examining microbial community diversity, emphasizing that organisms with exactly the same 16S rRNA gene sequence can exhibit very different ecological dynamics. Indeed, Salles et al. (2012) examined the links between rates of denitrification and phylogenies and highlighted the potential importance of horizontal gene transfer (HGT) by showing that similarity in *nirK* genes, which are thought to be subject to HGT, is not related to N_2_O accumulation rates. Furthermore, for *nirS* and 16S rRNA genes, Salles et al. (2012) showed that there was more explanatory power between structure and function at finer scales of phylogenetic resolution for denitrification and metabolic profiles respectively. Pearce et al. (2012) used metagenomics to examine a soil microbial community from Mars Oasis, Antarctica, and showed that while genera-level diversity was limited, species-level diversity was high. They proposed that this suggests strong selection on the types of taxa that can inhabit this extreme environment combined with high rates of diversification within those lineages. Related, Knope et al. (2012) used a microcosm approach to examine the importance of evolutionary history for diversification in bacteria. They showed that prior exposure to an environmental challenge led to higher rates of diversification. These studies suggest that understanding the coupling of ecological and evolutionary processes is key for interpreting microbial community patterns of structure and function.

Community assembly processes may also alter the relationship between “who's there” and “what they do” (Nemergut et al., 2013). Knope et al. (2012) found that arriving in a community first led to a greater degree of diversification within bacteria, likely because of niche-preemption. Pholchan et al. (2013) used a variety of manipulations to alter microbial community assembly in sludge reactors and showed that relationships between biodiversity and ecosystem function in these systems were unpredictable. They hypothesized that the relative importance of stochastic vs. deterministic assembly processes could change the relationship between biodiversity and ecosystem function. In their comment on the Pholchan manuscript, Knelman and Nemergut (2014) provide a conceptual framework illustrating how assembly, biodiversity and function may be related. Together, these studies provide growing evidence for the importance of assembly processes in determining microbial community properties.

Physical dynamics may also be key in regulating the relationship between structure and function. Schimel and Schaeffer (2012) propose a conceptual framework that highlights a requirement that biological processes need to be rate limiting or fate determining in order for community structure to matter for ecosystem function. For example, they propose that structure is not likely to be relevant for organic matter breakdown in mineral soils, where diffusion is limited and organic particles may be occluded or sorbed to soil surfaces. Likewise, Folse and Allison (2012) demonstrate that rates of diffusion of enzymes can affect community diversity and the relative proportion of generalists to specialists. Their work also showed high rates of diffusion coupled to high rates of production can lead to community bottlenecks and increases in stochasticity. As well, King et al. (2012) found that physical dynamics may also affect biotic relationships. They found that associations between plants and microbial community composition were less pronounced at higher elevations, likely due to an increase in the influence of physical harshness on community composition.

Together, the studies in this special issue highlight the role of a variety of ecological, evolutionary and physical dynamics in microbial community structure and function (Figure 1). This body of work emphasizes the importance of emergent, aggregate community properties and the role of community dynamics in variations in the strength of the structure-function relationships. As Schimel wrote in 1995 “At a small enough scale, microbial community structure must be a dominant control on ecological processes, but as we move up in scale toward the ecosystem and integrate across many individual communities, the influence of microbial community structures decreases.” Predicting when, where, how, and at what scale microbial communities may respond to environmental changes remains a research priority and these papers present new insights into this challenge.

**Figure 1:**
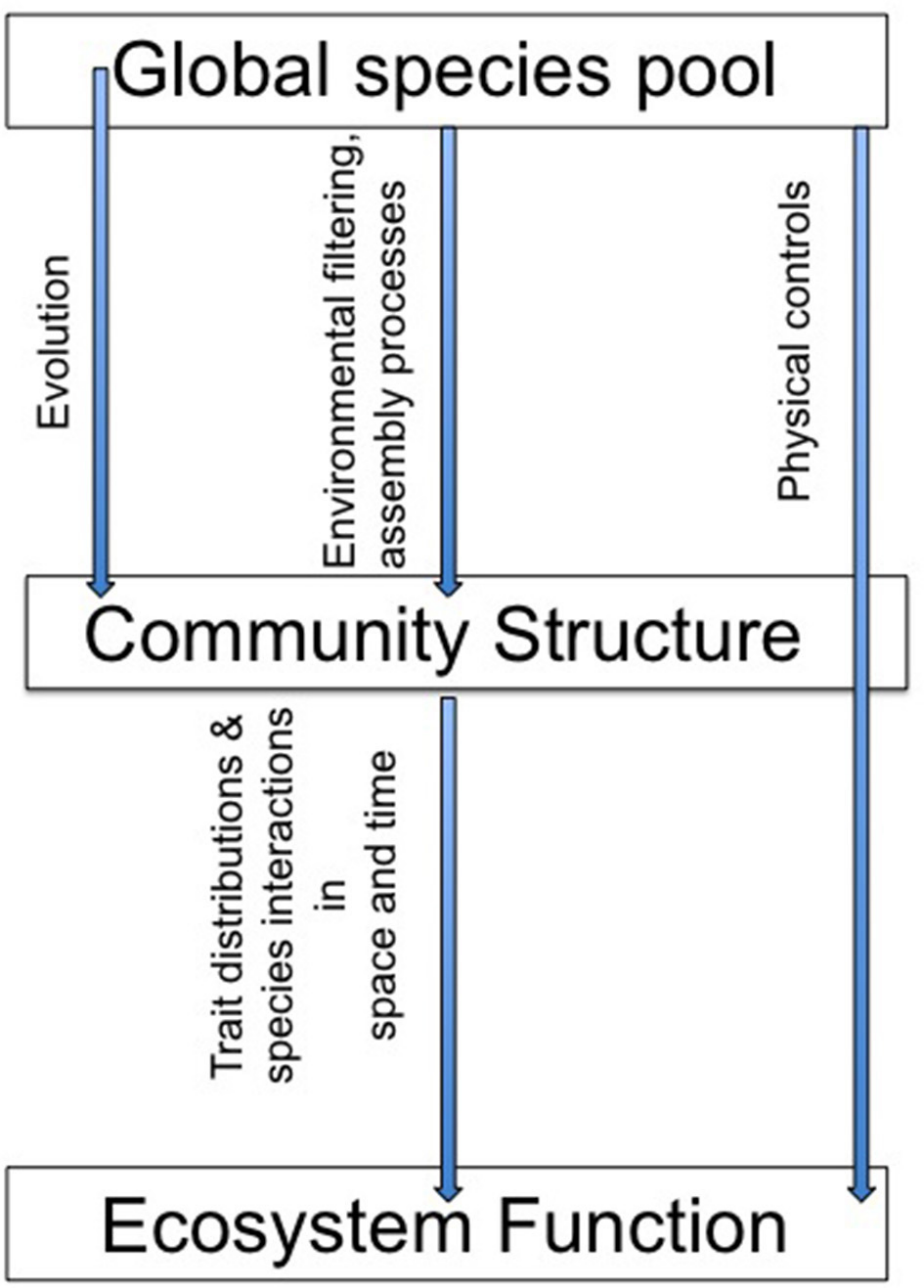
**Does “who's there” matter for “what they do”?** The papers in this special issue use modeling, empirical approaches, and literature reviews to address a suite of controls over the relationship between community structure and ecosystem function.

## Conflict of interest statement

The authors declare that the research was conducted in the absence of any commercial or financial relationships that could be construed as a potential conflict of interest.
